# Predictors of sentinel lymph node mapping failure in endometrial cancer: a retrospective multicenter study

**DOI:** 10.3389/fonc.2026.1799692

**Published:** 2026-04-07

**Authors:** Tomas Rokos, Marian Grendar, Jindrich Gobel, Lucia Kotulova, Erik Kudela, Terezia Pribulova, Erik Kozubik, Michal Kalman, Kamil Biringer

**Affiliations:** 1 Department of Gynecology and Obstetrics, Jessenius Faculty of Medicine in Martin Comenius University in Bratislava (JFMCU), Martin, Slovakia; 2 Laboratory of Bioinformatics and Biostatistics, Biomedical Center Martin (BIOMED), Jessenius Faculty of Medicine in Martin Comenius University in Bratislava (JFMCU), Martin, Slovakia; 3 Department of Obstetrics and Gynecology, Nemocnice Pardubického kraje a.s., Pardubice, Czechia; 4 Department of Pathological Anatomy, Jessenius Faculty of Medicine in Martin Comenius University in Bratislava (JFMCU), Martin, Slovakia

**Keywords:** endometrial cancer, ICG, predictive modeling, SLN biopsy, SLN mapping failure

## Abstract

**Objective:**

This study evaluated risk factors for indocyanine green (ICG) sentinel lymph node (SLN) mapping failure in endometrial cancer patients and assessed their value in predicting SLN mapping failure at the individual patient level.

**Methods:**

We conducted a retrospective study of endometrial cancer patients who underwent ICG-guided SLN biopsy. Risk factors for SLN mapping failure were identified using univariate and multivariate logistic regression analyses. Additionally, an imbalanced random forest (RF) algorithm was trained to predict SLN mapping success or failure. Predictive modeling was used to complement traditional statistical inference by translating population-level associations into patient-specific risk estimates.

**Results:**

A total of 190 patients were included, of whom 35 experienced SLN mapping failure. Univariate analyses showed that age 65 years and over (OR = 2.87, 95% CI 1.31–6.26; p = 0.007) and serous histological subtype (OR = 4.12, 95% CI 1.41–12.0; p = 0.006) were significantly associated with mapping failure. Multivariate analysis revealed that only age remained statistically significant (OR = 1.07 per year; 95% CI 1.02–1.13; p = 0.006). No significant associations were observed for obesity, morbid obesity, tumor grade, or FIGO stage. The discriminative ability of the RF model was poor (cross-validated AUC = 0.58, 95% CI 0.47–0.69).

**Conclusion:**

We suggest—on average—age 65 years and over and a serous histological subtype are risk factors for SLN mapping failure. However, the presence of these risk factors in an individual patient does not reliably predict SLN mapping failure. Additionally, more specific predictors may be needed for accurate individualized prediction.

## Introduction

Endometrial carcinoma was the sixth most common cancer among women worldwide in 2020, with more than 417,000 newly diagnosed cases and 97,000 cancer-related deaths (1). Together with cervical and ovarian cancer, endometrial cancer accounts for approximately one-third of newly diagnosed cancers in women worldwide (2).

Low-grade endometrioid carcinoma represents the most common histological subtype and is frequently diagnosed at an early stage, and is generally associated with a favorable prognosis (3). However, endometrial cancer represents a heterogeneous group of tumors with distinct prognostic characteristics. Tumor histology, grade, and lymphovascular space invasion are routinely assessed, whereas surgical staging is traditionally determined by the extent of local tumor spread and lymph node involvement. In addition, molecular classification has become essential for prognostic risk stratification and treatment planning (4).

Based on these clinicopathological and molecular characteristics, patients are stratified into risk groups that guide further management. In early-stage endometrial cancer, surgical treatment aims to remove macroscopic disease, detect occult metastases, and accurately stage the tumor to inform decisions regarding adjuvant therapy. Assessment of lymph node status remains a key component of surgical staging. For patients without radiological or intraoperative suspicion of extrauterine disease, sentinel lymph node (SLN) biopsy may be considered an alternative to systematic lymphadenectomy (5). In high-intermediate- and high-risk groups, where nodal assessment is recommended, systematic lymphadenectomy can be replaced by SLN biopsy (6).

Systematic lymphadenectomy is associated with several adverse effects, including lower-extremity lymphedema, lymphocyst formation, injury to surrounding structures, prolonged operative time, and increased blood loss (7). In contrast, SLN biopsy reduces operative time, the incidence of postoperative leg lymphedema (8), and intraoperative blood loss (9). Compared with systematic lymphadenectomy, SLN biopsy demonstrates at least comparable oncological outcomes, with a slightly higher pelvic lymph node detection rate and a marginally lower recurrence rate (10).

Despite these advantages, successful SLN mapping is not achieved in all patients, and failure of SLN detection may compromise the intended benefits of this approach. Identifying patient- and tumor-related factors associated with SLN mapping failure is therefore clinically relevant.

This study aimed to evaluate the effects of patient age, body mass index (BMI), and histopathological tumor characteristics on the success rate of SLN detection in patients with endometrial carcinoma.

## Materials and methods

This retrospective study included patients who underwent SLN mapping with indocyanine green (ICG) fluorescence imaging for endometrial cancer between January 2023 and August 2025. Patients were treated at the Clinic of Gynecology and Obstetrics, Jessenius Faculty of Medicine in Martin, Comenius University in Bratislava, and University Hospital Martin, Slovakia, and at the Pardubice Oncogynecology Center, Hospital of the Pardubice Region, Czechia. Inclusion criteria included histologically confirmed endometrial carcinoma, further managed surgically with SLN mapping. Collected data included patient age, type of surgical procedure, SLN detection rate and anatomical location, histopathological confirmation of SLNs and the presence of lymph node metastases, p53 status, mismatch protein repair (MMR) expression, FIGO stage, and body mass index (BMI). All demographic, clinical, operative, and pathological data were prospectively recorded in a clinical database, which served as the data source for this study.

During the surgical procedure, a total of 4 mL of ICG was injected cervically at the 3 and 9 o’clock positions; at each site, 1 mL was injected deep into the cervical stroma, and 1 mL was injected submucosally. Approximately 15 minutes after cervical injection, the retroperitoneal space opened, and near-infrared fluorescence imaging was utilized for real-time visualization of lymphatic pathways. A standardized tracer dose and injection technique were used in all patients. Successful bilateral SLN mapping was defined as visualization of at least one SLN in both hemipelves, whereas SLN mapping failure was defined as absence of SLN detection or unilateral SLN visualization.

Tumor histopathological evaluation and molecular testing were performed according to established guidelines (11, 12). SLNs were examined by dedicated pathologists using the ultrastaging technique described by Kim Ch. et al. (13). “Empty-packet dissection” was defined as the absence of lymph node tissue on pathological examination of a specimen submitted as an SLN.

For data analysis, exploratory data analysis (EDA) and univariate and multivariate statistical analyses were performed using jamovi (version 2.6) (14) and R (version 4.2.0) (15). Categorical variables were summarized as counts and percentages. For each predictor, contingency tables stratified by SLN mapping outcome (success vs. failure) were constructed and visualized using bar plots. Continuous variables (age and BMI) were summarized using descriptive statistics and visualized with boxplots stratified by SLN mapping outcome.

For each contingency table, Pearson’s χ² test of independence was applied after verification that expected cell counts met standard assumptions. Odds ratios (ORs) with 95% confidence intervals were calculated to quantify association in the univariate analyses. To evaluate the joint effect of predictors, a multivariate logistic regression model with a logit link function was fitted, with SLN mapping outcome as the dependent variable and age, BMI, tumor grade, and FIGO stage as independent variables. Adjusted ORs with 95% confidence intervals and corresponding p values were obtained. Multicollinearity was assessed using variance inflation factors (VIFs). Model fit was assessed using residual deviance, Akaike’s information criterion (AIC), and pseudo-R² statistics (McFadden, Cox–Snell, Nagelkerke, and Tjur), as well as an omnibus likelihood ratio test comparing the fitted model with the intercept-only model. Estimated marginal means for age and BMI were calculated using the emmeans package (16). Statistical analyses related to the random forest model were conducted in R (17). Graphical outputs were generated using the ggplot2 (18) and ggpubr (19) packages.

An imbalanced RF machine learning algorithm implemented in the randomForestSRC package (20) was trained on the entire dataset to predict SLN mapping outcomes (success vs. failure). Missing data (6.8% data points in the variable ‘grade’) were imputed using the impute.rfsrc() function. Out-of-bag (OOB) data were used to obtain unbiased estimates of predictive performance. Performance metrics, including sensitivity, specificity, negative predictive value (NPV), positive predictive value (PPV), accuracy, precision, recall, F-measure, and the Youden index, were calculated using the yardstick package (21). Partial dependence plots were generated to visualize the relationships between individual predictors and the OOB-predicted probability of SLN mapping success. Variable importance (VIMP) scores were used to rank predictors according to their contribution to the model. Finally, OOB density plots of class probabilities, OOB receiver operating characteristic (ROC) curves, and OOB calibration curves were generated to visually assess the model’s discriminative ability as well as the quality of its predictions from the calibration perspective.

Reports with details of data analyses are available at Mendeley repository, https://data.mendeley.com/datasets/ng5gdzvfz6/2

This is an observational study. The Research Ethics Committee with code IRB00005636 Jessenius Faculty of Medicine in MartinComenius University Bratislava IRB #1 has confirmed that no ethical approval is required.

## Results

The study included 190 patients with a median age of 64.0 years (range 34–85 years). Ninety-one patients (47.9%) were aged ≥65 years, while 99 patients (52.1%) were younger than 65 years. With respect to BMI, 64 patients (33.7%) were classified as nonobese (BMI <30 kg/m²), and 126 patients (66.3%) were classified as obese (BMI ≥30 kg/m²). The mean BMI was 32.9 ± 7.4 kg/m² (SD).

All patients underwent hysterectomy with SLN mapping using ICG. Laparotomy was performed in 4 primary planned patients, laparoscopy in 54 patients, and robotic surgery in 131 patients; in one patient, SLN biopsy was initially performed laparoscopically and subsequently converted to laparotomy due to large uterine myomas. According to definitive histopathological evaluation, the most common histological subtype was endometrioid carcinoma (n = 170), followed by serous carcinoma (n = 16), clear cell carcinoma (n = 3), and carcinosarcoma (n = 1).

The most common tumor grade was G2 (n = 105), followed by G1 (n = 49) and G3 (n = 25); tumor grade was not evaluated in 11 cases. Regarding molecular classification, p53 wild-type status was confirmed in 85 patients, p53 abnormality in 11, and p53 status was not evaluated in 94 patients. MMR-proficient tumors were identified in 63 patients, and MMR-deficient tumors in 23 patients; MMR status was not assessed in the remaining patients.

Sentinel lymph node metastases were identified in 5 patients, including isolated tumor cells in 2 patients. Owing to the high number of molecularly unclassified tumors, patients were staged according to the 2009 FIGO criteria as follows: FIGO I (n = 156), FIGO II (n = 18), FIGO III (n = 16), and FIGO IV (n = 0).

SLN mapping was successful in 155 patients, whereas bilateral intraoperative SLN mapping failure occurred in 19 patients, and unilateral failure in 16 patients. In SLN mapping failure cases was side-specific systematic lymphadenectomy or systematic lymphadenectomy performed, except one patient with unilateral mapping failure in whom systematic lymphadenectomy of hemiplevis was omitted according to operational terrain obscurity in clinical FIGO I stage. Among these patients no nodal metastases were identified. Empty-packet dissection was histologically confirmed in 15 patients, while in the Department of Obstetrics and Gynecology, Pardubice 5 empty packet dissection was confirmed (9,6%) and in the Department of Gynecology and Obstetrics, Jessenius Faculty of Medicine in Martin 10 packet dissection was confirmed (7,2%). Surgeries with empty packet dissection were performed by one surgeon in the Department of Obstetrics and Gynecology, Pardubice and two surgeons in the Department of Gynecology and Obstetrics, Jessenius Faculty of Medicine in Martin. There were no significant differences between surgeons, while all surgeons were of advanced learning curve. Patient characteristics are summarized in **Table 1**.

**Table 1:** Baseline patient characteristics according to SLN mapping outcome (N = 190).

Characteristic	Category	SLN mapping successful (n = 155)	SLN mapping failure^a^ (n = 35)
Age	< 65 years	88 (56.8%)	11 (31.4%)
	≥ 65 years	67 (43.2%)	24 (68.6%)
BMI	< 30 kg/m²	54 (34.8%)	10 (28.6%)
	≥ 30 kg/m²	101 (65.2%)	25 (71.4%)
Histological type	Endometrioid carcinoma	143 (92.3%)	27 (77.1%)
	Serous carcinoma	9 (5.8%)	7 (20.0%)
	Clear cell carcinoma	2 (1.2%)	1 (2.9%)
	Carcinosarcoma	1 (0.6%)	0 (0.0%)
Tumor grade	G1	40 (25.8%)	9 (25.7%)
	G2	91 (58.7%)	14 (40.0%)
	G3	20 (12.9%)	5 (14.3%)
	Not evaluated	4 (2.6%)	7 (20.0%)
p53 status	Wild-type	75 (48.4%)	10 (28.6%)
	Abnormal	10 (6.5%)	1 (2.9%)
	Not evaluated	70 (45.1%)	24 (68.6%)
MMR status	Proficient	57 (36.8%)	6 (17.1%)
	Deficient	19 (12.3%)	4 (11.4%)
	Not evaluated	79 (51.0%)	25 (71.4%)
FIGO stage (2009)	I	128 (82.6%)	28 (80.0%)
	II	13 (8.4%)	5 (14.3%)
	III	14 (9.0%)	2 (5.7%)
	IV	0 (0.0%)	0 (0.0%)

a SLN mapping failure include unilateral and bilateral mapping failures.

In univariate analysis, obesity (BMI ≥30 vs. <30 kg/m²), morbid obesity (BMI ≥40 vs. <40 kg/m²), FIGO stage (II–III vs. I), and tumor grade did not show clear differences between the SLN mapping success and failure groups. None of these variables were significantly associated with SLN mapping failure (all p values from Pearson’s χ² tests > 0.05).

**Figure 1:**
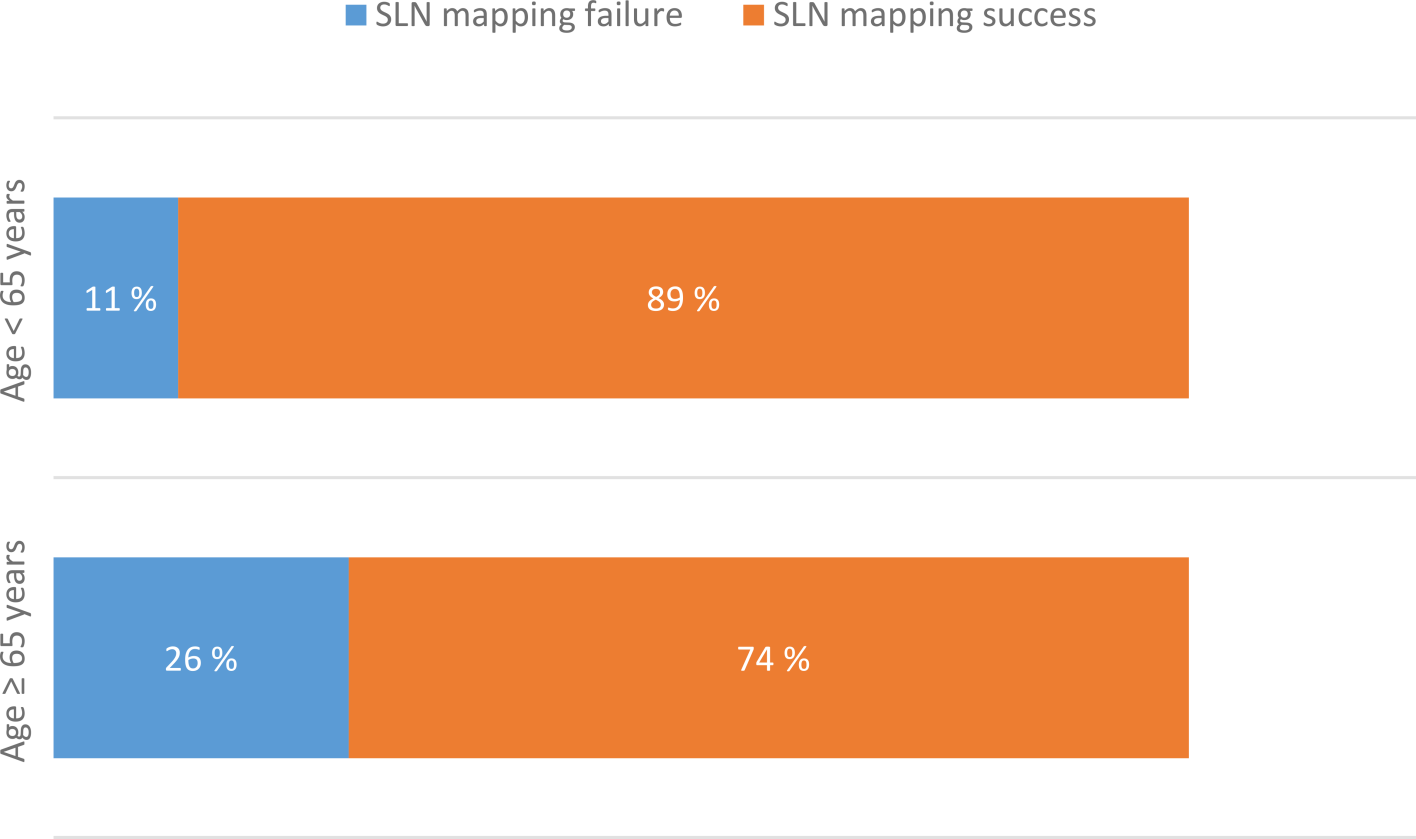
SLN mapping outcomes according to patient age (≥65 vs <65 years).

In contrast, patient age and histological subtype were associated with SLN mapping outcome. Among patients aged ≥65 years, SLN mapping failed in 24 of 91 patients (26.4%), compared with 11 of 99 patients (11.1%) younger than 65 years (**Figure 1**). This corresponded to significantly higher odds of SLN mapping failure in older patients (OR = 2.87, 95% CI 1.31–6.26; p = 0.007).

For histological subtype analysis, patients with clear cell carcinoma (n = 3) and carcinosarcoma (n = 1) were excluded due to the small sample size. When SLN mapping rates were assessed in endometrioid and serous subtypes, SLN mapping failed in 27 of 170 patients (15.9%) with endometrioid carcinoma and in 7 of 16 patients (43.8%) with serous carcinoma. This yielded a significantly higher odds ratio of SLN mapping failure in serous compared with endometrioid carcinoma (OR = 4.12, 95% CI 1.41–12.0; p = 0.006). However, the confidence interval was relatively wide, indicating limited precision of the estimate, and the magnitude of the association should therefore be interpreted with caution. For surgery type, SLN mapping failed in 24 of 131 patients (18.3%) undergoing surgery type 1, in 11 of 55 patients (20.0%) undergoing surgery type 2, and in 0 of 4 patients undergoing surgery type 3. There was no statistically significant association between surgery type and SLN mapping failure (Fisher’s exact test, p > 0.9).

These analyses considered each predictor separately and did not account for potential confounding by other covariates; therefore, the observed associations were further examined in a multivariate logistic regression model.

In the multivariate logistic regression model with SLN mapping outcome (success vs. failure) as the dependent variable, associations with age, BMI, tumor grade, and FIGO stage were evaluated. Although histological subtype showed modest collinearity with other predictors (VIF = 2), it was excluded from the final model primarily due to sparse data patterns that resulted in model instability and near separation. Sparse data pattern was reason for excluding also type of surgery from the predictors. The analysis was based on complete cases with available tumor grade data (n = 179). A summary of the multivariate logistic regression model is presented in **Table 2**.

**Table 2:** Multivariate logistic regression model for SLN mapping outcomes.

Predictor	Estimate	SE	Z	P	Odds ratio	95% confidence interval	
						Lower	Upper
Intercept	-6.6906	2.0479	-3.267	0.001	0.00124	2.24e-05	0.0688
Age ^a^	0.0704	0.0255	2.755	0.006	1.0729	1.0205	1.128
BMI ^a^	0.0277	0.0303	0.914	0.361	1.02809	0.9688	1.091
Grade 2 vs. 1	-0.6211	0.4981	-1.247	0.212	0.53733	0.2024	1.4264
Grade 3 vs. 1	0.1956	0.7487	0.261	0.794	1.216	0.2803	5.2755
FIGO II+III vs. I	-0.9898	0.7566	-1.308	0.191	0.37163	0.0843	1.6374

a Odds ratios for age and BMI are expressed per unit increase. SE – standard Error, Z – Z score, P – p value.

**Figure 2:**
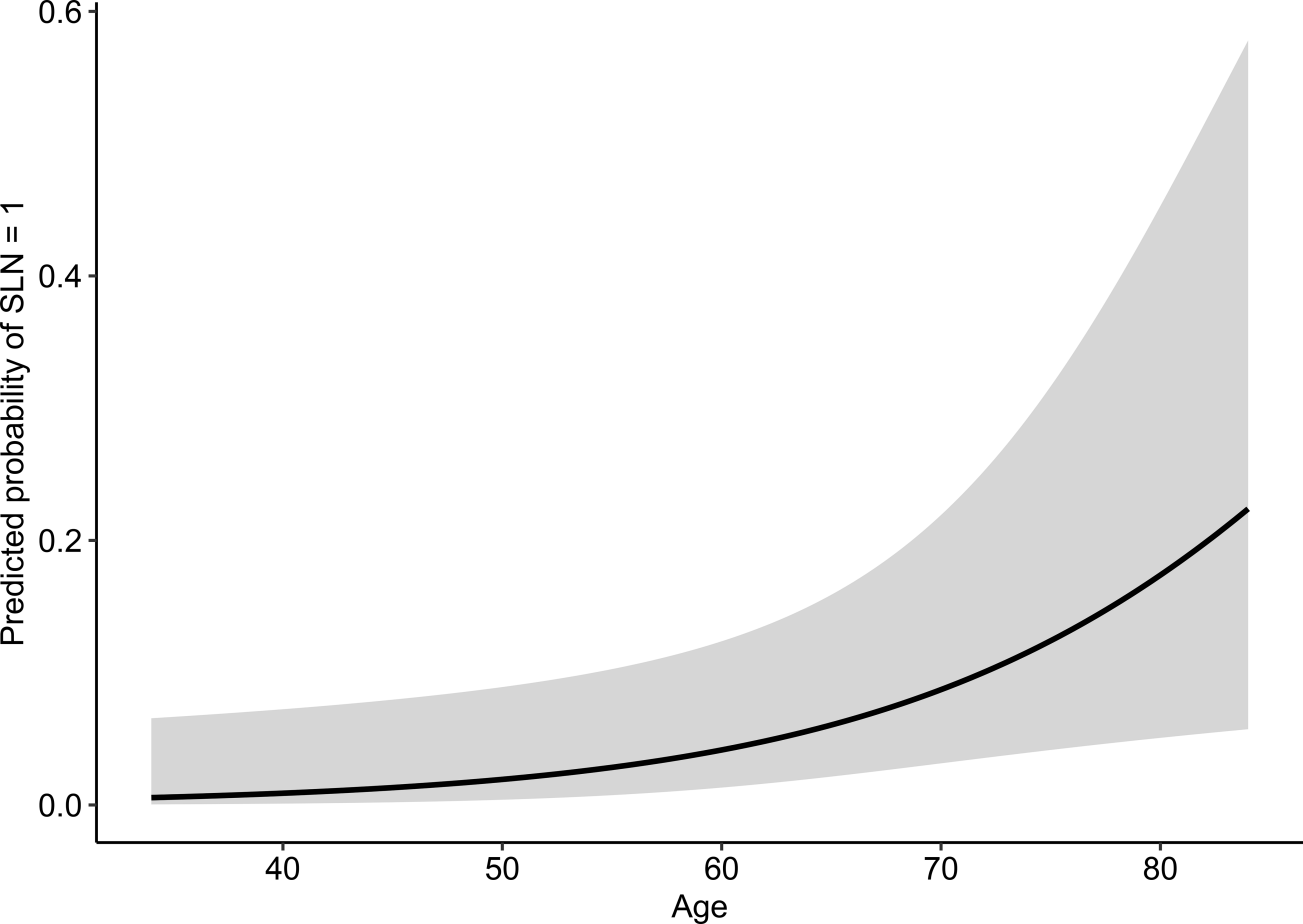
Model-based predicted probability of SLN mapping failure according to age.

After adjustment for BMI, tumor grade, and FIGO stage, age remained the only variable significantly associated with SLN mapping outcome. The odds of SLN mapping failure increased by approximately 7% per additional year of age (OR = 1.07, 95% CI 1.02–1.13; p = 0.006), consistent with the increasing model-based estimated probability of failure across age (estimated marginal means from the logistic regression model; **Figure 2**).

In contrast, BMI, tumor grade, and FIGO stage were not significantly associated with SLN mapping status (BMI: OR = 1.03; 95% CI 0.97–1.09; p = 0.361; G2 vs. G1: OR = 0.54; 95% CI 0.20–1.43; p = 0.212; G3 vs. G1: OR = 1.22; 95% CI 0.28–5.28; p = 0.794; FIGO II–III vs. FIGO I: OR = 0.37; 95% CI 0.08–1.64; p = 0.191; **Table 2**). These results describe adjusted associations but do not directly quantify the predictive performance of the covariates or the model. To assess predictive ability, a separate random forest model was constructed and evaluated.

Sensitivity analyses comparing the analysis including all failure cases (unilateral and bilateral) with the analysis restricted to bilateral failures showed consistent results. Histological type remained significantly associated with failure in both approaches, while age ≥65 years was significant only in the analysis including all failures. Differences observed for surgery type were mainly related to changes in category structure and sample size.

In multivariate logistic models, age remained the only variable significantly associated with failure in both analyses, with similar effect estimates. Other variables were not statistically significant, and both models demonstrated low explanatory power (Tjur’s R² ≈ 0.05–0.07).

**Figure 3:**
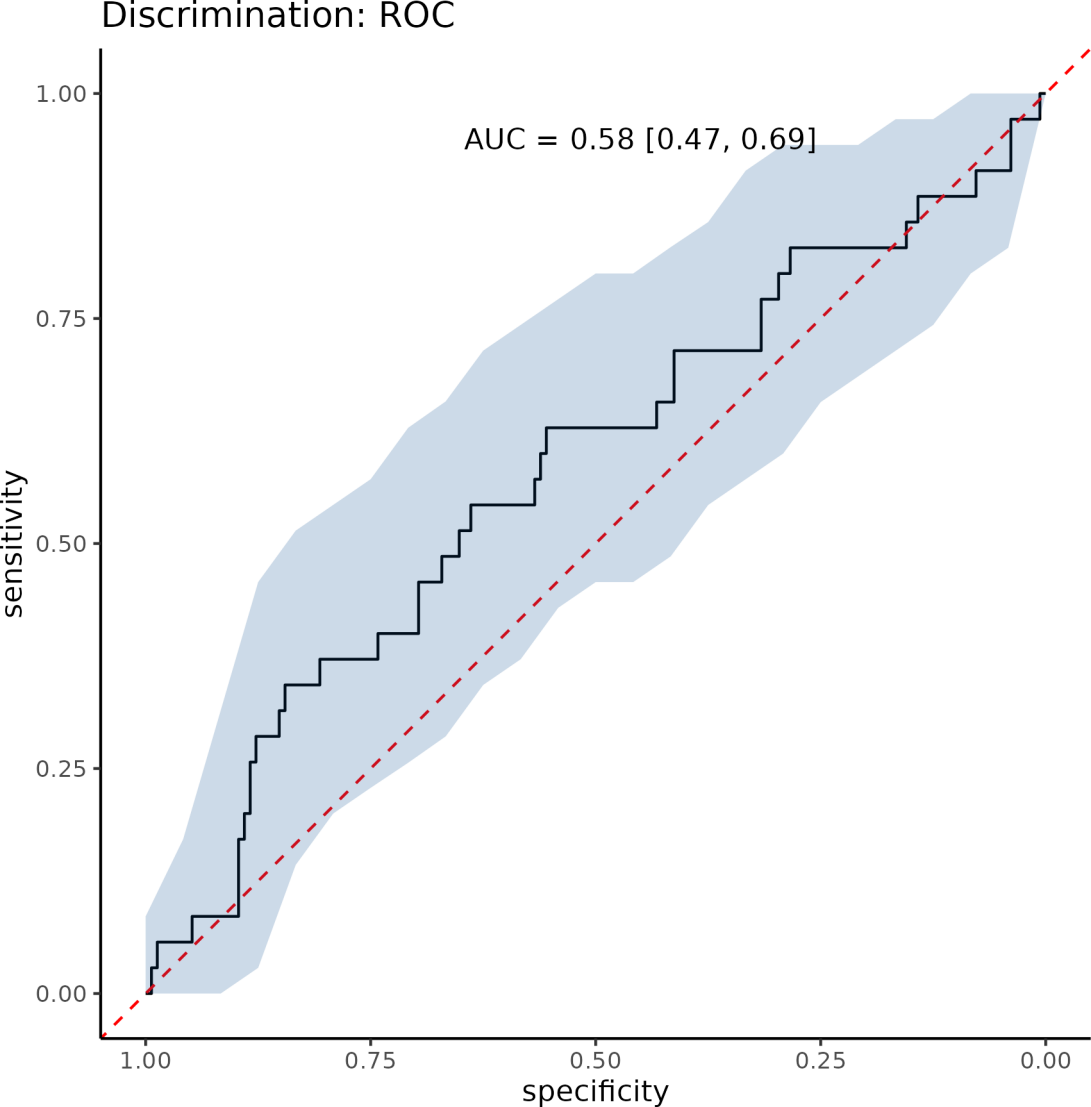
Variable importance plot of Random Forest. Predictors (age, histological subtype, tumor grade, FIGO stage, BMI and type of surgery) are ranked on the y-axis from the most-important at the top. The higher the value on the x-axis, the more important is a predictor. Negative importance implies the predictor do not contribute in predicting the class.

In the machine learning analysis, the Random Forest algorithm identified patient age as the most influential predictor of SLN mapping failure (**Figure 3**), followed by histological type. Grade, BMI, type of surgery and FIGO stage were found not useful for predicting SLN failure.

**Figure 4:**
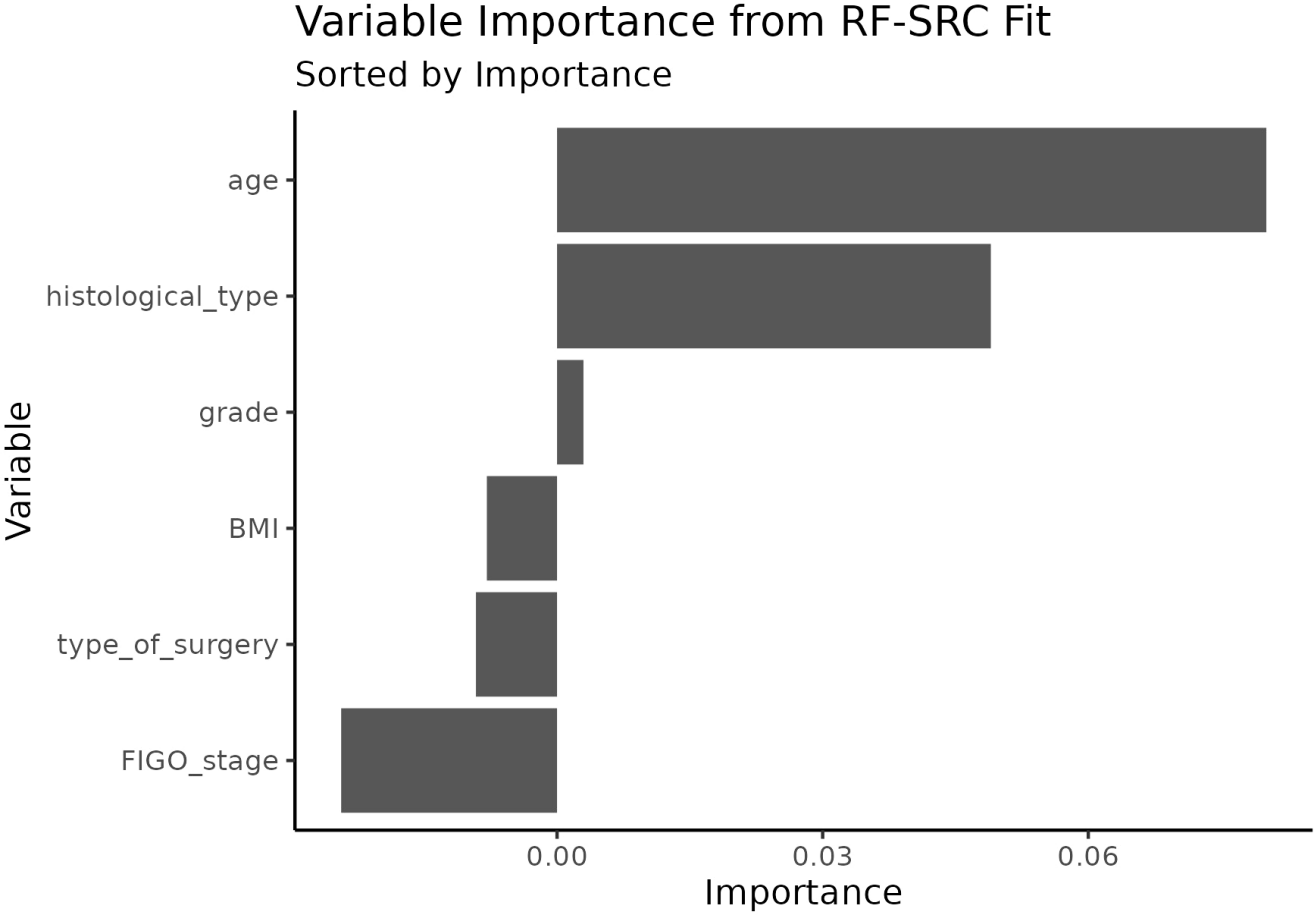
Out-of-bag (OOB) receiver operating characteristic (ROC) curve of the random forest model.

The overall discriminative performance of the model was poor, with an OOB AUC of 0.58 (**Figure 4**). This finding indicates that although age is significantly associated with SLN mapping failure at the population level, it has limited ability to accurately discriminate between individual patients who will or will not experience mapping failure. In practical terms, older patients tend to have a slightly higher modeled risk on average. Nevertheless, substantial overlap exists between the predicted risk distributions of patients with successful and failed SLN mapping. Thus, the effect of age, while statistically significant, is too weak and inconsistent to support clinically useful individualized prediction.

To express the predictive performance of the random forest model in clinically interpretable terms, discrimination was translated into the predictive odds ratio (ORpred) derived from the confusion matrix and the observed prevalence of SLN mapping failure in the dataset, which was estimated at 0.18. Using the optimal classification threshold, the model yielded an ORpred of 3, indicating that patients predicted to experience SLN mapping failure had approximately 3-fold higher odds of actual failure than those predicted not to fail. This metric was used solely to aid clinical interpretability and does not imply adequate model performance. Although the odds ratio exceeds unity, it reflects only modest discriminative ability, consistent with the low AUC (0.58), and confirms that the model performs poorly in distinguishing individual patient outcomes.

## Discussion

Aging is a progressive and irreversible process characterized by a gradual decline in cellular and tissue function across all organ systems (22) and is accompanied by degeneration and remodeling of the immune system, a phenomenon known as immunosenescence. Changes in the function and structure of the lymphatic system are also involved in the aging process and are associated with an increased risk of lymphatic-related disorders such as lymphedema, inflammation, and cancer (23). Degeneration of the lymphatic system can be quantified numerically, as the human body contains approximately 300–500 lymph nodes, and aging is associated with a reduction in their number (24). In addition to numerical decline, age-related lymph node changes include reduced size, calcification, lipomatous atrophy, and fibrosis with microarchitectural remodeling, which, through destruction of the reticular stroma, may lead to deterioration of lymph node function (25). Moreover, the contractile frequency of lymphatic vessels decreases with age due to reduced smooth muscle cell coverage, while glycocalyx components, intercellular junction molecules, and extracellular matrix elements decline in the lymphatic vessels of older individuals (26). Finally, aged collecting lymphatic vessels become dilated, exhibit reduced contractility, and show increased permeability (27), which may impair effective lymphatic drainage.

These age-related changes are likely to influence SLN mapping performance, which is consistent with our findings. Univariate analysis revealed a significantly greater likelihood of SLN mapping failure in patients aged ≥65 years than in younger patients (OR = 2.87, 95% CI 1.31–6.26; p = 0.007). These results are in accordance with those of the SAGE study, which identified older age as a risk factor for SLN mapping failure in patients with endometrial cancer, with failure rates of 22.9% in patients younger than 65 years and 33.2% in patients aged ≥65 years (28). Similarly, in multivariate analysis, Giudici A. et al. (29) reported that age ≥65 years was the only independent predictor of SLN mapping failure (OR = 2.2, 95% CI 1.3–3.8; p = 0.003), whereas other clinical, surgical, and pathological factors were not significant. That study also demonstrated lower bilateral SLN detection rates in older patients (85%) than in younger patients (93%) (29).

In our cohort, 91 patients were aged ≥65 years, and SLN mapping failed in 24 (26.4%) of them, whereas among 99 patients younger than 65 years, SLN mapping failed in 11 (11.1%). Our results, therefore, confirm a statistically significant association between age ≥65 years and SLN mapping failure, which persisted in multivariate analysis. Moreover, the odds of SLN mapping failure increased by approximately 7% per additional year of age (OR = 1.07, 95% CI 1.02–1.13; p = 0.006). Comparable findings were reported by Bretova P. et al. (30), who also identified age as a significant risk factor for bilateral SLN mapping failure in multivariable analysis (30).

Aging is closely linked to chronic low-grade inflammation (“inflammaging”), which is considered a key contributor to age-related diseases (23). Moreover, reactive nitrogen and oxygen species, including nitric oxide, as well as cytokines can damage lymphatic endothelial cells and suppress their proliferation, leading to lymphatic dysfunction. Lymphangiogenic growth factors, particularly vascular endothelial growth factor C (VEGF-C) and VEGF-D, and their primary receptor VEGFR-3, play a central role in lymphatic endothelial cell proliferation, differentiation, and survival (31). Inflammatory states may also increase lymphatic vessel permeability, with VEGF-A acting as a critical mediator (32). With advancing age, the synthesis of angiogenic cytokines such as VEGF, platelet-derived growth factor (PDGF), and TGF-β decreases, resulting in impaired angiogenesis and lymphangiogenesis (33). Although VEGF-C overexpression may promote lymphangiogenesis, excessive or dysregulated VEGF-C signaling can paradoxically impair lymphatic function (34).

Obesity represents another condition associated with lymphatic dysfunction. Reduced VEGFR-3 transcription has been linked to obesity, potentially contributing to impaired lymphatic function (31). A meta-analysis by Zafar M. I. et al. (35) revealed increased VEGF-B and VEGF-C expression in obese individuals (35), which may reflect compensatory responses to reduced VEGFR-3 signaling. Obesity is also characterized by chronic inflammation. Although the exact mechanisms are not fully understood, chemokines such as C-C motif chemokine ligand 21 (CCL21) have been proposed to mediate perilymphatic inflammatory cell clustering (36). Several studies, including those by Insalaco G. et al. (37) and the ObeLyX (38), reported a statistically significant association between higher BMI and SLN mapping failure. In contrast, our results did not demonstrate a significant association between obesity (BMI ≥30 kg/m²) and SLN mapping failure compared with patients with a BMI <30 kg/m² (OR = 1.03, 95% CI 0.97–1.09; p = 0.361). Similarly, although Johnson L. et al. (39) reported lower SLN mapping success rates in morbidly obese patients (39), we observed no clear differences in SLN mapping success or failure among patients with a BMI ≥40 kg/m².

In addition to SLN mapping failure, we also evaluated empty-packet dissection. The ObeLyX study reported significantly higher odds of empty-packet dissection in obese women than in those with a BMI <30 kg/m² (8.2% vs. 3.9%, p = 0.022) (38). In our cohort, empty-packet dissection was confirmed in 15 patients: 13 with a BMI ≥30 kg/m² and 2 with a BMI <30 kg/m², suggesting a similar trend. However, formal statistical significance could not be established.

Tumor-related factors may further disrupt lymphatic drainage. Excessive VEGF-C expression by tumor cells can induce lymphatic sinus hyperplasia, thereby altering lymphatic flow even before nodal metastases become apparent, potentially leading to aberrant tracer migration, bypass of the true sentinel lymph node, or dissemination of the tracer to multiple nodes (40, 41). Advanced-stage tumors may invade lymphovascular spaces, disrupt lymphatic anatomy, and cause nodal metastases, all of which may adversely affect SLN mapping (42, 43). Raffone A. et al. (44) demonstrated significantly increased odds of SLN mapping failure in patients with FIGO stage III–IV disease (OR = 1.89; p = 0.01) in a systematic review and meta-analysis (44). In contrast, we did not observe a significant association between FIGO stage and SLN mapping failure (FIGO II–III vs. I: OR = 0.37, 95% CI 0.08–1.64; p = 0.191), likely reflecting the relatively small sample size and the absence of FIGO stage IV patients in our cohort. Similarly, Taliento C. et al. (45) found no significant association between FIGO stage and SLN mapping failure but identified a significant association with non-endometrioid histology (45), which is consistent with our findings.

The aggressive features of serous endometrial carcinoma, including a high incidence of lymphovascular space invasion and deep myometrial invasion, may disrupt lymphatic drainage pathways and alter tracer migration from the cervix to regional lymph nodes (42, 45). In our study, SLN mapping failed in 15.9% of endometrioid tumors and in 43.8% of serous tumors, and serous histology was significantly associated with SLN mapping failure (OR = 4.12, 95% CI 1.41–12.0; p = 0.006). However, tumor grade was not significantly associated with SLN mapping failure, in line with findings reported by Raffone A. et al. (44).

Despite the established advantages of SLN biopsy over systematic lymphadenectomy, a subset of patients continues to experience SLN mapping failure. Identifying prognostic factors for such failure may aid preoperative planning and individualized surgical decision-making, which are key principles of personalized medicine. While molecular alterations such as PTEN, PIK3CA, CTNNB1, and TP53 mutations, microsatellite instability (MSI), and MMR deficiency serve as predictive biomarkers for targeted therapies (46), no clinically applicable predictive model for preoperative identification of SLN mapping failure currently exists.

In our study, age ≥65 years was consistently associated with SLN mapping failure in both univariate and multivariate analyses, consistent with previous reports (29, 30, 47). Age was identified as the most important predictor of SLN mapping failure also by the Random Forest algorithm, by its Variable Importance (VIMP) algorithm for ranking predictors. The only other important predictor identified by RF was the histological type. Grade, BMI, type of surgery and FIGO stage provided no discriminative value for predicting SLN mapping failure. The overall discriminative performance of the predictors was poor (AUC = 0.58). The predictive performance, expressed in epidemiological terms as Predictive Odds Ratio (ORpred) was ORpred = 3, indicating moderate associations. However, in the context of prediction, such effect sizes correspond to sensitivity and specificity values of approximately 0.6, reflecting limited discriminative ability. Clinically useful discrimination (sensitivity and specificity approximately 0.9) would require odds ratios on the order of 80–100, which far exceed those observed in our study. While no published studies have reported odds ratios of this magnitude for SLN mapping failure, several predictive models for lymph node metastases have achieved AUC values ≥0.7 by incorporating multiple preoperative and pathological factors, such as deep myometrial invasion, CA-125 levels, aberrant p53 expression, MSI status (48), pelvic lymph node enlargement, progesterone receptor expression, and histological subtype (49).

In our study was SLN mapping failure rate 43,8% in serous endometrial carcinoma patients, what raises the question of whether initiating surgical staging with an attempt at SLN mapping is clinically justified. Despite high-grade histology, some serous endometrial cancer tumors can be still classified as the low-risk or intermediate risk tumors according to the tissue invasion (confined to the uterine corpus or to the uterus), lymphovascular space invasion and molecular classification. According to the current guidelines in case of SLN mapping failure side-specific systematic lymphadenectomy should be done for patients at high–intermediate or high risk, and can be considered in patients at presumed intermediate risk (50). It means that despite high-grade histology, there are some patients in whom SLN biopsy may not be mandatory. What is more, in all patients with presumed uterus-confined disease sentinel lymph node biopsy should be done for staging purposes (50). These procedure did not increase recurrence or mortality compared to systemic lymphadenectomy (51). Moreover, in comparison of no lymphadecentomy versus versus pelvic lymphadenectomy, no lymphadenectomy likely reduces lymphocyst formation and results in a large reduction in lymphedema (52). Although we pointed out in serous carcinoma histology SLN mapping failure in almost one half patients, we suggest no primary systematic lymphadenectomy indicated preoperatively in these patients, since according to the current evidence SLN biopsy should be the first step procedure in disease staging. Primary indicated systematic lymphadenectomy would unnecessarily increase morbidity and can negatively affect quality of life. Preoperatively known higher risk of SLN mapping failure can be partially balanced by optimizing preoperative preparation including planning minimally invasive approach, surgery performed or supervised by experienced surgeon with ≥20 endometrial cancer cases per year, ICG use to SLN mapping (42), and also adherence to the standardized algorithms (44).

Further research is needed to identify additional predictors and improve individualized risk stratification for SLN mapping failure in patients with endometrial cancer. The development of a predictive model based on preoperatively available variables could assist in selecting the most appropriate primary surgical approach and identifying patients in whom systematic lymphadenectomy should be planned upfront rather than attempting SLN mapping in the setting of a high predicted risk of failure. Importantly, SLN mapping failure is influenced not only by patient- and tumor-related factors but also by technical variables, including imaging equipment, surgeon experience, injection site, depth, timing, and ICG dose or concentration.

While our study offers several strengths, the limitations of this work include absence of comprehensive molecular characterization, which is a major limitation of the study. According to missing molecular classification FIGO 2023 classification could not be applied in line with the current guidelines. Another limitation is exclusion of histological subtype from the final model according to the reason mentioned in the results section.

The results of this study suggest that older age (in both univariate and multivariate analyses) and serous histological subtype (in univariate analysis) are associated with an increased risk of SLN mapping failure in patients with endometrial cancer. Importantly, statistical significance does not necessarily translate into clinically useful predictive performance, and the identified associations were insufficient to accurately predict SLN mapping failure at the individual–patient level. The presence of risk factors, especially their multiple presence, increases the risk of mapping failure, which indicates the appropriateness of compensating factors that, on the contrary, increase the success rate of SLN detection, for example, the use of ICG mapping with its correct use, a minimally invasive approach, or the surgery performed by a surgeon with a high learning curve (42). Future research should focus on enhancing personalized surgical planning and optimizing operative strategies for patients at risk of SLN mapping failure.

## Data Availability

The datasets presented in this study can be found in online repositories. The names of the repository/repositories and accession number(s) can be found in the article/supplementary material.
